# Canine parvovirus induces G1/S cell cycle arrest that involves EGFR Tyr1086 phosphorylation

**DOI:** 10.1080/21505594.2020.1814091

**Published:** 2020-09-02

**Authors:** Xiaofeng Dai, Xuanhao Zhang, Yujie Miao, Peiyu Han, Jianying Zhang

**Affiliations:** a Wuxi School of Medicine, Jiangnan University, Wuxi, China; b The First Affiliated Hospital of Xi’an Jiaotong University, Xi’an, China; c School of Biotechnology, Jiangnan University, Wuxi, China; d Henan Academy of Medical and Pharmaceutical Sciences, Zhengzhou University, Zhengzhou, Henan, China; e Department of Biological Sciences, University of Texas at El Paso, El Paso, TX, USA

**Keywords:** Canine parvovirus, cell cycle arrest, EGFR, virotherapy

## Abstract

Canine parvovirus (CPV) has been used in cancer control as a drug delivery vehicle or anti-tumor reagent due to its multiple natural advantages. However, potential host cell cycle arrest induced by virus infection may impose a big challenge to CPV associated cancer control as it could prevent host cancer cells from undergoing cell lysis and foster them regain viability once the virotherapy was ceased. To explore CPV-induced cell cycle arrest and the underlying mechanism toward improved virotherapeutic design, we focus on epidermal growth factor receptor (EGFR), a cellular receptor interacting with TfR that mediates CPV-host interactions, and alterations on its tyrosine phosphorylation sites in response to CPV infection. We found that CPV could trigger host G1/S cell cycle arrest via the EGFR (Y1086)/p27 and EGFR (Y1068)/STAT3/cyclin D1 axes, and EGFR inhibitor could not reverse this process. Our results contribute to our understandings on the mechanism of CPV-induced host cellular response and can be used in the onco-therapeutic design utilizing CPV by preventing host cancer cells from entering cell cycle arrest.

## Introduction

Some viruses have natural affinities for receptors specifically expressed on tumor cells and thus have been combined with nanotechnology for tumor targeting [1]. Due to enhanced Fenton effect, iron accumulation is elevated in rapidly dividing cancerous cells that leads to upregulated transferrin (iron-binding blood plasma glycoprotein) receptor (TfR) expression [2]. TfRs were reported to be over-expressed in a variety of tumor cells such as glioma cells [3], breast cancer cells [4], colon carcinoma cells [5], erythroleukemia cells [6] and pancreatic cancer cells [7]. Thus, tagging a drug or image contrast agent to TfRs for tumor-specific delivery had been proposed as a promising onco-therapeutic strategy. Canine parvovirus (CPV) utilizes TfRs for binding and cell entry into canine and human cells, rendering them a potential nanotool in medicine. Toward this goal, Singh, et al. expressed the CPV-VP2 capsid protein in a baculovirus expression system, namely CPV virus-like particles (CPV-VLPs), and used it for anti-tumor drug delivery [1].

Besides, parvoviruses have been used as an antitumor agent and immune cell activator. For instance, parvovirus H-1 (H-1PV) has been utilized in oncolytic virotherapy that utilizes lytic viruses to kill tumor cells without infecting normal cells [8]. Lysis of cancer cells releases tumor-associated antigens that induce anticancer immunity and thus can be considered as a cancer vaccine. This type of oncotherapy is advantageous in preventing tumor relapse and metastasis due to immunological memory [9].

Despite the promising roles of parvoviruses in cancer control, they were reported to induce cell cycle arrest [10–14]. Cell cycle checkpoints function as the precise temporal controller of cell replication cycles that are regulated by differential combinations of cyclins and cyclin-dependent kinases (CDKs) [15]. Aberrant external or internal perturbations including virus infection may result in cell cycle checkpoint arrest that provides time for cells to recover before proceeding to the next cell cycle stage [16]. Increasing evidence has suggested that a cellular DNA damage response triggered by an invading single-stranded parvoviral genome could induce cell cycle arrest in parvovirus-infected cells to create a favorable environment for viruses to complete their lifecycles [14]. Therefore, understanding the critical events involved in parvovirus-induced cell cycle arrest and exploring approaches to combat against such events to occur would be expected to largely improve the onco-therapeutic efficacies of virotherapies.

EGFR was reported to regulate TfR-mediated iron homeostasis through interacting with TfR in cytosol and maintaining TfR expression on cell surface in non-small cell lung cancers [17]. Here we take CPV as the model of parvoviruses to explore the mechanism driving CPV-induced cell cycle arrest with a focus on EGFR-mediated signaling to aid in parvovirus-mediated virotherapies for cancer control.

## Materials and methods

### Cells and viruses

Madin-Darby canine kidney (MDCK) cells and Crandall feline kidney (CRFK) cells were purchased from Chinese Academy of Sciences Cell Bank, and cultured in RPMI medium that contains 10% fetal bovine serum (FBS) and 1% penicillin-streptomycin solution. CPV was obtained from Da Bei Nong Group, China, and propagated using CRFK and MDCK cells.

### Infection of MDCK cells and CRFK cells

MDCK and CRFK cells were grown to 80%-90% confluence following CPV infection. Cells were harvested at 12 h, 24 h, 36 h, 48 h after infection. The median tissue culture infective dose (TCID50) was measured as the virus dilution folds where 50% of the cells were infected through plating cells in a 96-well plate, inoculating cells with a 10-gradient sequential diluted solution of viral fluid at a step-size of 10 folds, and monitoring cells for 5 to 7 d until 50% cells showed cytopathic effect (CPE). The Reed-Muench approach was used to calculate TCID50 by $log_{10}TCID50=\left(A-0.5\right)/\left(B-0.5\right)\times \left(A-B\right)+A$, where A and B each represents the fraction of cells showing CPE bigger and smaller than 0.5, respectively, and within 0 and 1. Multiplicity of infection (MOI) was computed by MOI=0.7×TCID50×Virus volume/Cellamount.

### Primer design

The primers were designed using the Primer 5 software according to the *VP2* gene of the CPV DNA sequence (A26575.1) in GenBank and synthesized in Suzhou Genewiz Biotechnology Co., Ltd. Polymerase Chain Reaction (PCR) was performed to select the optimal primers using CPV nucleic acid as the template and PrimerStar Max Mix (Takara Biomedical Technology Co., Ltd.) as the amplification kit. The primers designed were listed in Table 1, and the length of the detected virus DNA fragment was 1575 bp.

**Table 1.** Primers used for virus detection in real-time PCR.

Primers name	Primers sequence
Real-time PCR-CPV-F	5ʹ-CATTGGGCTTACCACCATTT-3’
Real-time PCR-CPV-R	5ʹ-AAATGGCCCTTGTGTAGACG-3’

### Standard curve construction

Virus DNA was extracted using PureLink™ Pro 96 Viral RNA/DNA Purification Kit (ThermoFisher Scientific, USA) and reverse transcribed into cDNA. The target fragment was obtained through PCR using primers designed in Table 1 and PrimerStarMax (Takara Bio, Japan). The DNA fragment was ligated with pGEM®-T Easy Vector (Promega, China) using T4 DNA ligase (Takara Bio, Japan). The concentration of the constructed plasmid was measured using Nanophotometer-N50 (Implen, Germany), and diluted into eight gradients at a step size of 10 using Tris-EDTA buffer solution. The copy number at each concentration was computed using $Copy\;number=6.2\times 10^{23}\times Plasmid\;concentration\;f/\left(base\;pair\;number\times 660\right)$. Cycle threshold (Ct) values were obtained for each plasmid concentration using real-time PCR. The standard curve was constructed by fitting the data into a linear regression.

### PCR

Cells were cultivated in 6-well plates, with 1×10^6^ cells/well. Cells were inoculated with 100 μL virus. TCID50 was 10^7.4^ and MOI was 1.75.Infected cell culture medium was used as the template. A 10 μL system was used that included 5 μL PrimerStar Max Mix, 0.5 μL of forward and backward primers, 2 μL of template, and 2 μL ddH_2_O. The reaction condition was: pre-denaturation at 98°C for 10 s, 95°C for 10 s, 60°C for 30 s, and 72°C for 20 s, for 40 cycles.

### Real-time PCR

Cells were cultivated in 6-well plates, with each well containing 1 million cells. A 100 μL of virus was used to inoculate cells, with TCID50 being 10^7.4^ and MOI being 1.75. Cells were cultured 48 h after virus inoculation, and supernatants were collected as the sample following the manufacture’s protocol of UltraSYBR MixTure Kit (CW0957M, Cwbio Co. Ltd.). Here, 4 μL samples, 10 μL 2×UltraSYBR MixTure, 1 μL forward and 1 μL backward primers (Table 1), 4 μL ddH_2_O were mixed and centrifuged before running the real-time PCR program (pre-denaturation at 95°C for 10 min; 95°C for 10 sec, 60°C for 1 min, 72°C for 20 sec, for 40 cycles) in Roche LightCycler 480 real-time PCR. Each sample has three replicates.

### Cell counting

Cells were cultivated in 96-well plates, 5 million cells/well. A 10 μL of virus was added, with TCID50 being 10^7.4^ and MOI being 3.5. Cells were grown to 80%-90% confluence. Cells were infected by CPV virus following CCK8 (purchased from MedChemExpress) viability detection at 12 h, 24 h, 36 h, and 48 h, respectively. The standard adheres to the cell absorbance value that represents the cell activity. Each sample has five replicates.

### Western blot

Cells were cultivated in T25 flasks, 3×10^6^ cells/flask. A 500 μL of virus was added to cells with TCID50 being 10^7.4^ and MOI being 2.93. Cells were washed twice using PBS and lysed in RIPA lysis buffer supplemented with protease inhibitors for 20 min on ice and centrifuged at 12,000 g for 20 min before supernatant collection. The protein concentration was estimated using the BCA Protein Assay Kit (Beyotime). Proteins (50 ug) per lane were resolved by SDS-PAGE and transferred to PVDF membrane. After blocking with 5% skim milk powder in TBS plus Tween-20 buffer, the membrane was incubated using the appropriate primary antibodies at 4ºC overnight followed by secondary antibody incubation for 2 h at room temperature. Antibody binding was visualized by developing the blot using enhanced chemiluminescence reagent. The bands were visualized using OmegaLumG (UVP) followed by Image J software analysis. The total protein of the infected MDCK and CRFK cells was extracted, separated using an 8% SDS-PAGE gel at 110 V for 70 min, and transferred from the SDS-PAGE gel to the PVDF membrane using a film transfer device. The primary antibody was added following incubation at 4°C for 8–12 h and TBST washing. The secondary antibody was supplemented and left standstill at the room temperature for 1–2 h, following TBST cleaning and gel imager detection. Each assay was repeated three times.

Primary antibodies used included antibodies against cyclin D1 (catalog number: 55,506 T), p27 (catalog number: 3688S), EGFR (catalog number: 2085S), pEGFR (Y1086) phosphorylation (catalog number: 2220S), pEGFR (Y1068) phosphorylation (catalog number: 3777S), pEGFR (Y1148) phosphorylation (catalog number: 4404S), STAT3 (catalog number: 9139 T), pSTAT3 (Y705) phosphorylation (catalog number: 9145 T), GAPDH (catalog number: 5174 T), and secondary antibodies include anti-mouse IgG/HRP-linked antibody (catalog number: 7076 T) and anti-rabbit IgG/HRP-linked antibody (catalog number: 7074 T). All antibodies were purchased from Cell Signaling Technology, Inc. All antibodies were diluted 1:1000 for primary antibodies and 1:5000 for secondary antibodies before usage following the manufacture’s protocol.

### Flow cytometry

Cells were cultivated in T25 flasks, with 3×10^6^ cells per flask. A 500 μL of virus was added, with TCID50 being 10^7.4^ and MOI being 2.93. Cells were grown in 6-well plates, washed using PBS and digested with EDTA-free trypsin, centrifuged at 1000 rpm/min for 5 min following supernatant removal to retain the pellets. Cell pellets were washed using 500 μL of PBS, centrifuged at 1000 rpm/min for 5 min, and the supernatants were removed to retain the pellet. Cell pellets were resuspended in 70% ethanol and placed in a 4°C refrigerator overnight for fixation. Fixed cells were centrifuged at 1000 rpm/min for 5 min to remove the supernatants, and cell pellets were suspended in 500 μl PBS, added with 5 μl of Propidium iodide (purchased from Beyotime) staining agent, and mixed on ice for 30 min. Cell cycle detection was performed using a BD Accuri C6 flow cytometer, and data analysis was performed using Flowjo software.

### Cell membrane potential detection assay

Cells were cultivated in 96-well plates, 1×10^5^ cells/well. A 10 μL of virus was added to cells with TCID50 being 10^7.4^ and MOI being 1.75. Cells were grown in 96-well plates following CPV synchronous inoculation (viruses were inoculated before cell bottom adhesion) and 2.5% FBS medium cultivation for 48 h. DMSO was used to dissolve Bis (1,3-dibutylbarbituric acid) trimethine oxonol (catalog number: D8189, Sigma-Aldrich (Shanghai) Co., Ltd.) at a final concentration of 1 mM. Cell medium was replaced by 100 μL cell culture medium containing 5 μL Bis trimethine per well following incubation at 37°C, 5% CO_2_ for 1 h. Cell membrane potential was measured using a multifunctional microplate reader (Synergy ™ H4, BioTek), where the excitation and emission wavelengths were set at 388 nm and 418 nm, respectively. Data were analyzed using Gene5 software. Each sample has five replicates.

### Ca^2+^ flux detection assay

Cells were cultivated in 96-well plates, 1×10^5^ cells/well. A 5 μL of virus was added to cells with TCID50 being 10^7.4^ and MOI being 1. Cells were grown in 96-well plates following CPV synchronous inoculation and 2.5% FBS medium cultivation for 48 h. DMSO was used to dissolve Fura-2 AM (catalog number: S1052, Beyotime Biotechnology Co., Ltd.) at a final concentration of 1 mM. Cell medium was replaced by 100 μL cell culture medium containing 5 μL Fura-2 AM per well following incubation at 37°C, 5% CO_2_ for 30 min. Calcium level was detected using a multifunctional microplate reader (Synergy ™ H4, BioTek), where the excitation and emission wavelengths were set at 340 nm and 510 nm, respectively. Data were analyzed using Gene5 software. Each sample has five replicates.

### Statistical test

Two-way two-sample T-test with equal variance was used to assess the statistical significance.

## Results

### CPV infection reduces cell viability in CRFK and MDCK cells through halting cells at the G1/S phase

**Figure 1.**
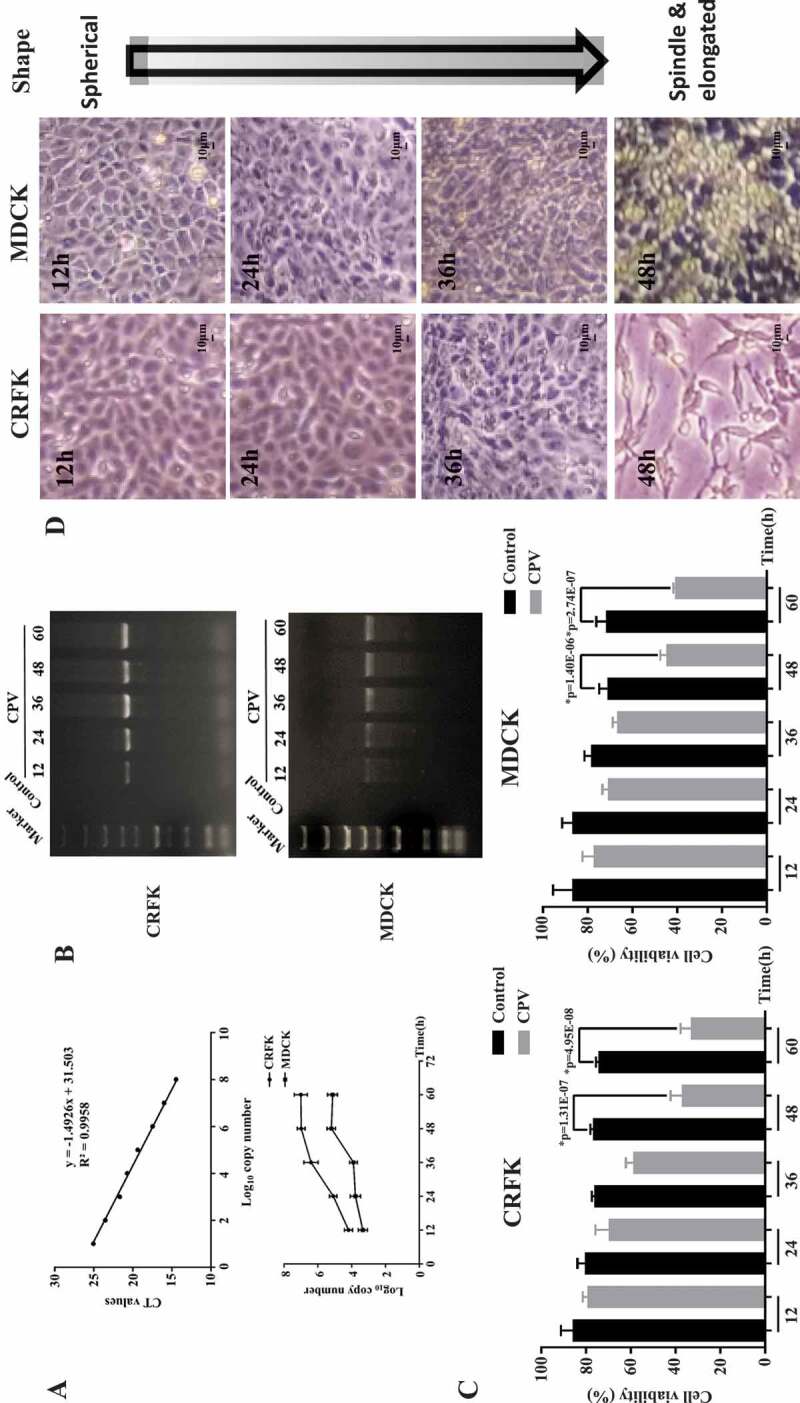
Virus titration, host cell viability and morphology at differential time intervals after CPV infection. (a) Standard curve and virus titration as detected using qPCR in CRFK and MDCK cells. (b) Virus titration as detected using PCR gel electrophoresis in CRFK and MDCK cells. (c) Host cell viabilities after CPV infection in CRFK and MDCK cells. (d) Host cell morphological images on CPV infection for MDCK and CRFK cells. The viral gene used for virus quantification was ‘*VP2ʹ.*

The real-time PCR, PCR, and CCK8 assays were conducted to examine virus amount, host cell viability, and morphology at different time intervals after CPV infection. Viral nucleic acid rather than viral titer was used to quantify virus amount as viral titer often involves cytopathic effect that does not solely and accurately reflect virus amount. Virus amount increased (Figure 1(a,b)) and cells’ viability decreased (Figure 1(c)) on CPV invasion in both CRFK and MDCK cells, and such an alteration exhibited a duration-dependent pattern. The maximum inhibition on cells’ viability was reached at 48 hours following a plateau after virus infection (p = 1.31E-7 in CRFK cells, p = 1.40E-6 in MDCK cells) in our tested time durations and experimental setting. Cells’ morphology changed from a spherical to a spindle and elongated shape with the duration following CPV infection (Figure 1(d)).

**Figure 2.**
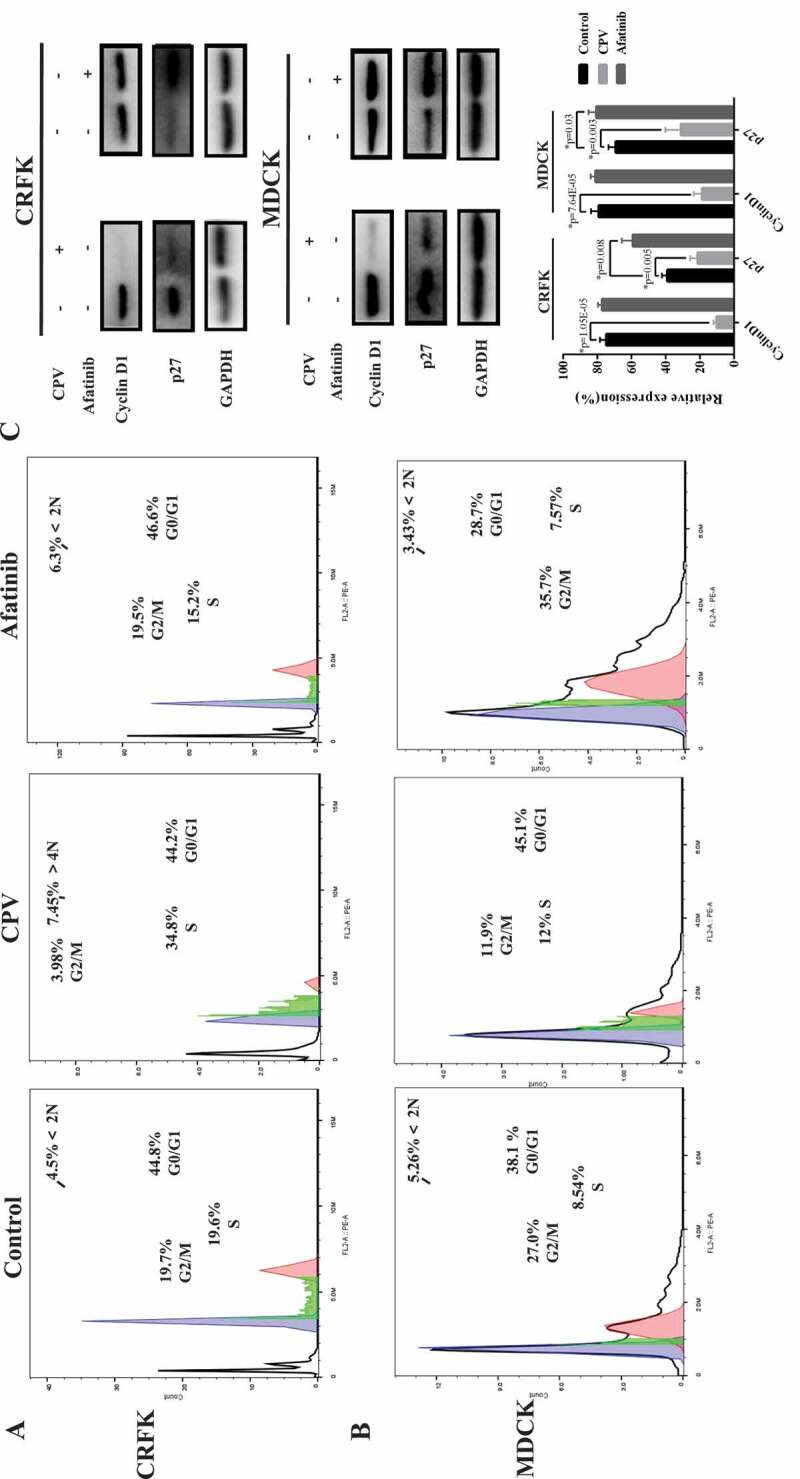
CPV-induced G1/S cell cycle arrest. Flow cytometry images showing CVP-induced G1/S cell cycle arrest in (a) CRFK and (b) MDCK cells. (c) Expression of key G1/S cell cycle arrest-related proteins. Each experiment was repeated 3 times, with one being shown in this figure. The pie chart was constructed by taking an average of each percentage from all repeats. Quantification results were prepared by normalizing cyclin D1 and p27 expression by GAPDH expression.

Flow cytometry was performed to study how CPV infection affects host cell cycle. The S phase considerably increased on CPV infection in both CRFK and MDCK cells (Figure 2(a,b)). Specifically, the S phase increased from 19.7% to 34.8% in CRFK cells (p = 0.045), and from 8.54% to 21% in MDCK cells (p = 0.036). The cell cycle profile was slightly reversely modified on Afatinib (an EGFR inhibitor) exposure in both CRFK and MDCK cells as compared with CPV infection (Figure 2(a,b)). In particular, the S phase dropped from 19.6% to 15.2% in CRFK cells, and from 8.54% to 7.57% in MDCK cells.

**Figure 3.**
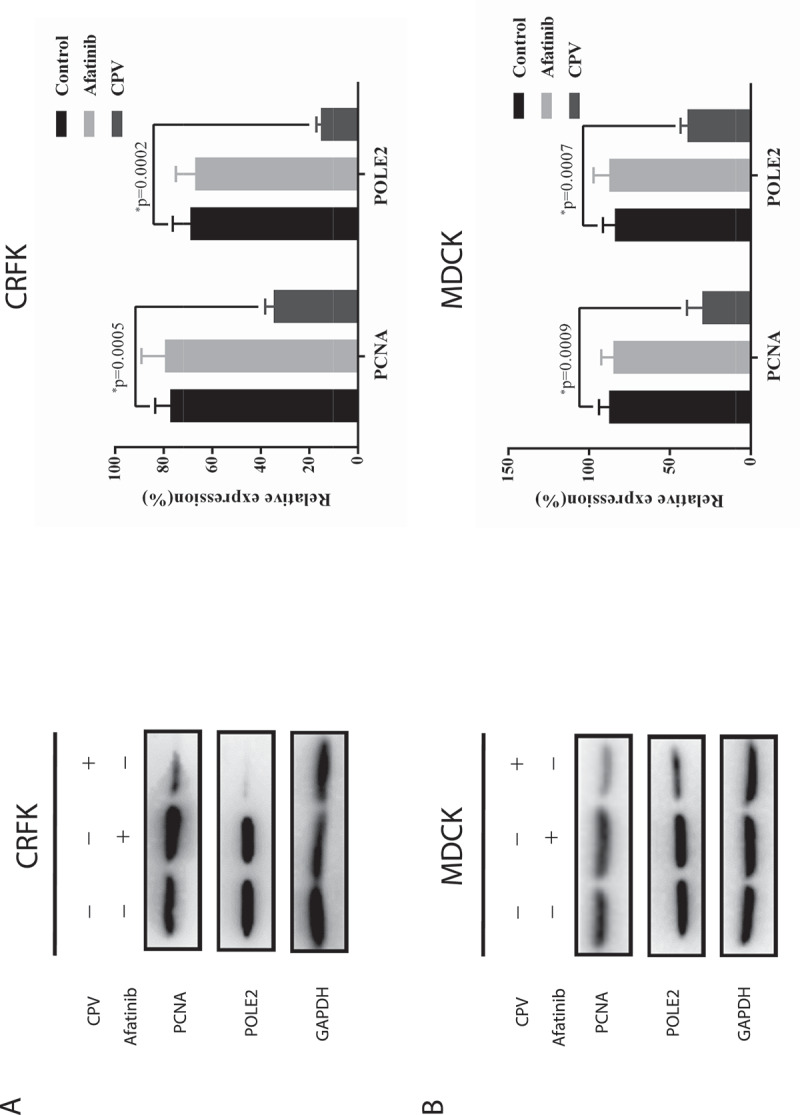
Expression of proteins relevant to DNA replication and cell proliferation on CPV infection or Afatinib exposure in (a) CRFK cells and (b) MDCK cells. Quantification results were prepared by normalizing PCNA and POLE2 expression by GAPDH expression.

Western blot was conducted to show how the expressions of proteins related to cell cycle arrest, DNA replication, and cell proliferation were modified after CPV infection. Among the measured proteins involved in the cell cycle, p27 expression was reduced on CPV infection (p = 0.005 in CRFK, p = 0.003 in MDCK) and increased on Afatinib exposure (p = 0.008 in CRFK, p = 0.03 in MDCK), both with statistical significance (Figure 2(c)); the level of cyclin D1 was reduced on CPV infection with statistical significance (p = 1.05E-05 in CRFK, p = 7.64E-05 in MDCK) but was unaffected when being treated with Afatinib (Figure 2(c)). The expression of PCNA and POLE2, two proteins involved in DNA replication and cell proliferation [18,19], showed similar profiles as cyclin D1 on CPV and Afatinib treatment (Figure 3). That is, significantly reduced expression of PCNA (p = 5E-04 in CRFK, p = 9E-04 in MDCK) and POLE2 (p = 2E-04 in CRFK, p = 7E-04 in MDCK) was observed on CPV infection but not on Afatinib treatment.

**Figure 4.**
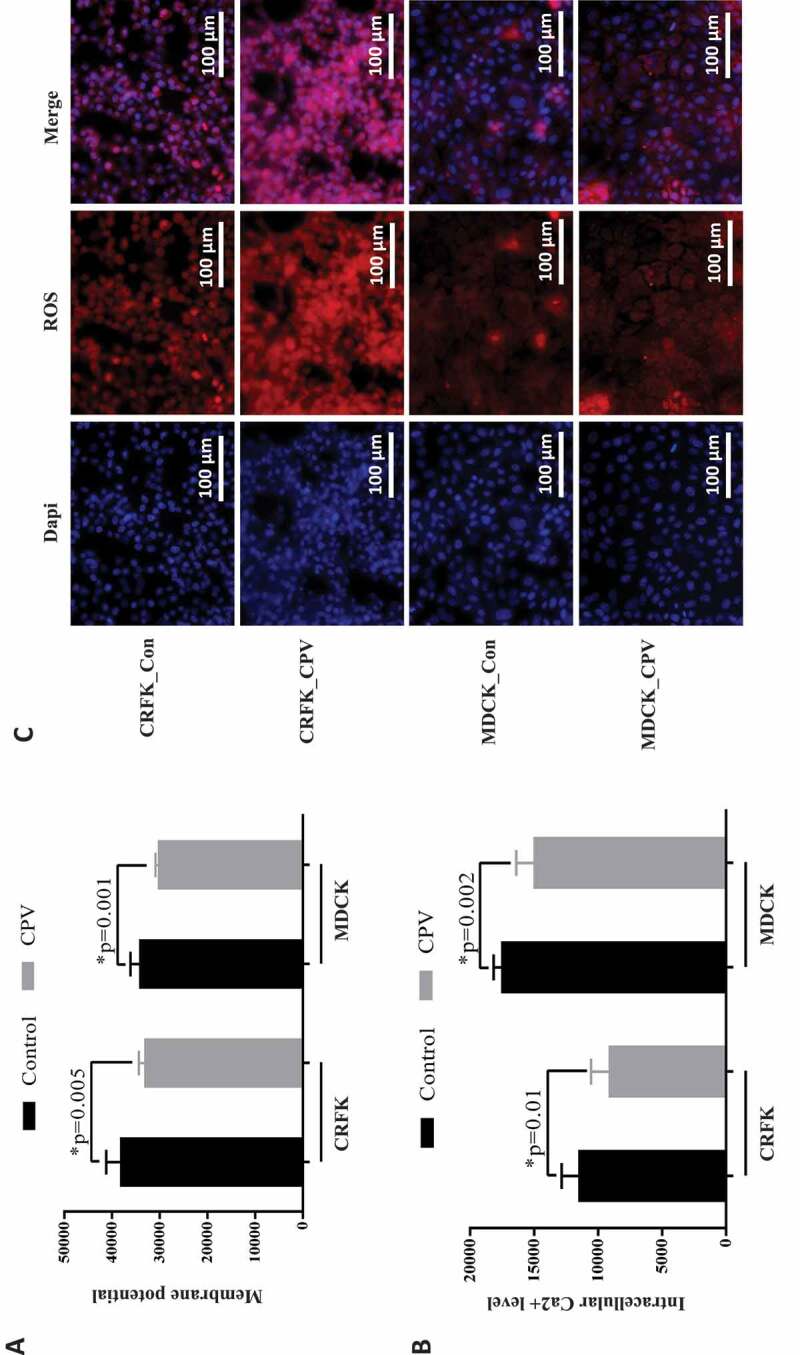
Cellular response to CPV infection. (a) Membrane potential, (b) intracellular Ca^2+^ level, and (c) intracellular ROS level on CPV infection.

In addition, membrane potential, intracellular Ca^2+^ and reactive oxygen species (ROS) were monitored before and after CPV infection. The results showed that CPV infection significantly decreased the membrane potential (Figure 4(a)), intracellular level of Ca^2+^ (Figure 4(b)) and increased intracellular ROS level (Figure 4(c)) in CRFK (p = 0.005 for membrane potential, p = 0.01for Ca^2+^) and MDCK (p = 0.001 for membrane potential, p = 0.002 for Ca^2+^) cells.

### EGFR (Y1086) phosphorylation and STAT3 signaling are involved in CPV-induced G1/S cell cycle arrest

**Figure 5.**
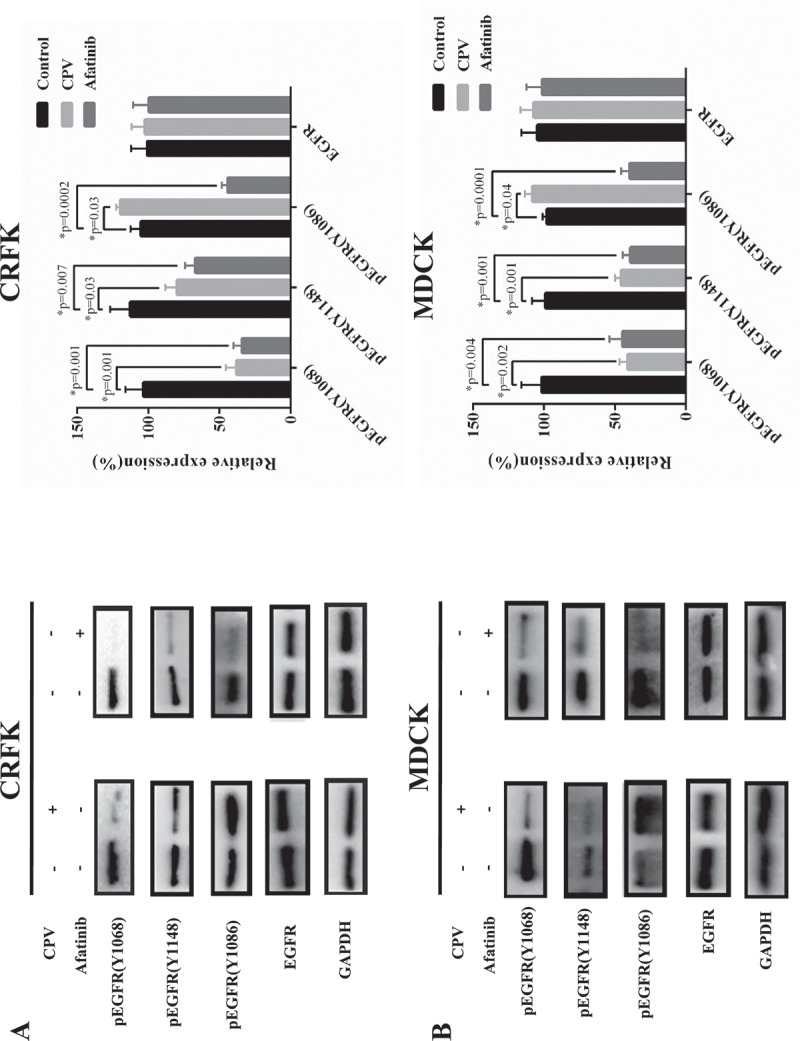
EGFR total protein expression level and phosphorylation status on CPV infection. Expression of EGFR protein and EGFR phosphorylation status at Y1086, Y1068, Y1148 on CPV infection and being treated with Afatinib in (a) CRFK cells and (b) MDCK cells. Afatinib is an EGFR tyrosine phosphorylation inhibitor. Quantification results were prepared by normalizing EGFR expression and phosphorylation by GAPDH expression.

We examined the protein expression level of EGFR and STAT3 as well as their phosphorylation status at multiple sites to explore altered cell signaling on CPV infection. Among the three tyrosine phosphorylation sites located in the cytoplasmic domain of EGFR (i.e. Y1068, Y1148, Y1086), Y1086 phosphorylation was the sole site that was considerably increased on CPV infection and showed a reverse pattern by adding Afatinib in CRFK (Figure 5(a)) and MDCK (Figure 5(b)) cells. We examined the immunofluorescence of these three EGFR phosphorylation sites on CPV invasion, and observed clearly increased expression of EGFR (Y1086) and slightly decreased phosphorylation at EGFR (Y1086) and EGFR (Y1148) sites in both CRFK and MDCK cells (supplementary Figure 2–4). In addition, we observed linear association between virus titration and EGFR (Y1086) phosphorylation (Figure 7(a)).

**Figure 6.**
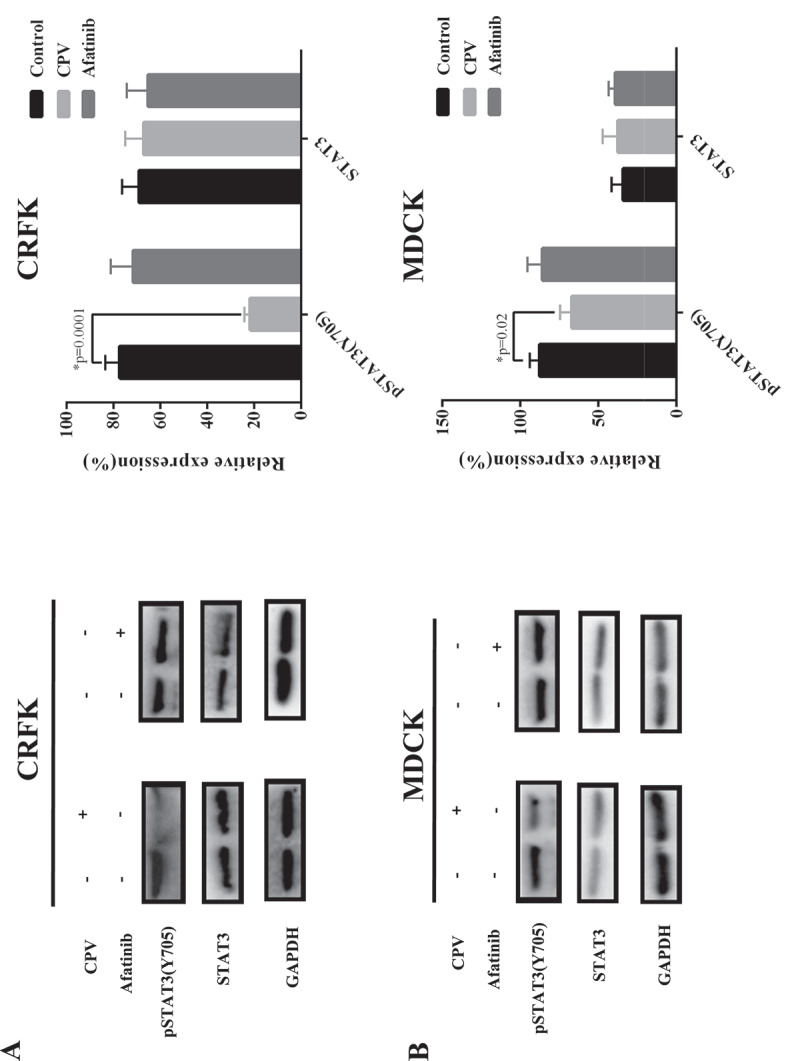
STAT3 total protein expression level and phosphorylation status on CPV infection. Expression of STAT3 protein and STAT3 phosphorylation status at Y705 on CPV infection and being treated with Afatinib in (a) CRFK cells and (b) MDCK cells. Afatinib is an EGFR tyrosine phosphorylation inhibitor. Quantification results were prepared by normalizing STAT3 expression and phosphorylation by GAPDH expression.

STAT3 (Y705) phosphorylation level was significantly reduced on CPV infection in both CRFK (p = 0.001, Figure 6(a)) and MDCK cells (p = 0.02, Figure 6(b)), suggesting the suppressive role of CPV on STAT3 signaling. Supplementing cells with Afatinib did not significantly affect STAT3 total and phosphorylation levels (Figure 6(a,b)), implying that STAT3 (Y705) de-phosphorylation was not mediated by EGFR (Y1086) phosphorylation.

## Discussion

We found from this study that virus multiplication increased with a sacrifice of reduced cell viability, and the host cell cycle was arrested at the G1/S stage. The significantly extended S phase provided viruses with ample DNA and protein resources to achieve rapid replication. In consistent with this, we observed reduced intracellular Ca^2+^ level and cell membrane potential (Figure 4(a,b)), suggesting decreased Ca^2+^ influx and halted G1 progression [20–22]. The observed distinct cell morphology alteration, i.e. cells became fibroblast-like on CPV infection, may possibly be caused by the cell cycle arrest, cell dedifferentiation, cytopathic effect, or other cell programs that require further explorations.

The p27 protein prevents cells from exiting the G1 and entering the S stage through binding to and preventing the activation of cyclin D/CDK4 or cyclin E/CDK2 complexes [23]. Both p27 expression and EGFR (Y1086) de-phosphorylation were reduced on CPV infection and increased on Afatinib exposure, suggesting that EGFR (Y1086) phosphorylation was associated with p27 suppression that contributed to cell accumulation at the S phase. Such an association with p27 was not found in EGFR (Y1068) and EGFR (Y1148).

**Figure 7.**
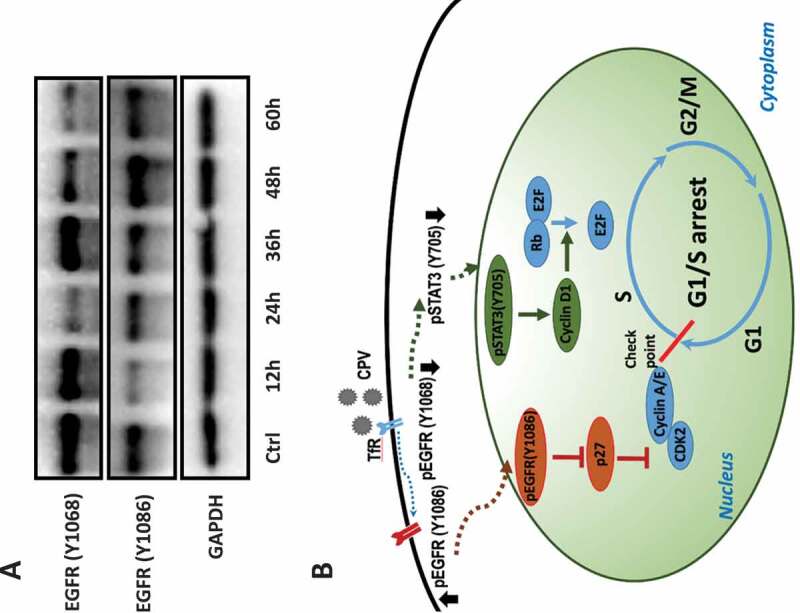
Conceptual scheme of CPV-triggered G1/S cell cycle arrest and kinetics of EGFR activation throughout virus life cycle. (a) Western blots showing EGFR phosphorylation status at Y1086 and Y1068 at different time points after CPV infection in CRFK cells. (b) Conceptual scheme of CPV-triggered G1/S cell cycle arrest via the EGFR(Y1086)/p27 and STAT3(Y705)/cyclin D1 axes. CPV binds to TfR on infection that triggers EGFR (Y1086) phosphorylation and EGFR (Y1068) de-phosphorylation. Phosphorylated EGFR (Y1086) is translocated to the nucleus where it contributes to reduced p27 expression; dephosphorylated EGFR (Y1068) is associated with reduced STAT3 (Y705) phosphorylation and decreased cyclin D1. Reduced p27 and cyclin D1 expression ultimately leads to cell cycle arrest at the G1/S stage.

Cyclin D1 promotes the G1/S transition through releasing E2F from the E2F/Rb complex [24]; and PCNA and POLE2 impose cellular replication stress once repressed as a result of EGFR alteration [19]. Concomitantly reduced expression of these genes and STAT3 (Y705) phosphorylation on CPV infection but not Afatinib exposure suggested a positive regulatory role of STAT3 on cyclin D1, PCNA, and POLE2, and that such a relationship was not solely dependent on the EGFR (Y1086) phosphorylation. Indeed, STAT3 activation was reported to be induced by EGFR (Y1068) phosphorylation [25,26], and EGFR (Y1068) was suppressed on CPV infection (Figure 5(a)). Thus, CPV-induced G1/S cell cycle arrest may involve both EGFR (Y1086) phosphorylation and EGFR (Y1068) de-phosphorylation. Indeed, EGFR (Y1086) and EGFR (Y1068) exhibited an increased and a decreased profile with time, respectively, after CPV infection (Figure 7(a)), which are consistent with the increased patterns on virus titer and viral gene expression under this condition (Figure 1(a,b)).

To this end, we would say that enhanced EGFR (Y1086)/p27 and reduced STAT3/cyclin D1 signaling are both indispensable to achieve significant G1/S cell cycle arrest (Figure 7(b)). In consistent with this, we observed dramatic G1/S cell cycle arrest on CPV infection but only slightly decreased S phase percentage (almost comparable with the control) on Afatinib exposure. This also suggests that EGFR phosphorylation inhibitors are not sufficient to function as a reverse operator of CPV on host G1/S cell cycle arrest.

Suppression on STAT3 phosphorylation and EGFR/Akt/cyclin D1 signaling was reported to jointly contribute to cell cycle arrest in glioblastoma cells [27]. We found that STAT3 phosphorylation and cyclin D1 expression were all suppressed on CPV infection which is similar to the anti-cancer mechanism reported in [27], indicative of the natural advantage of CPV in being used as the delivery vehicle of drugs against cancers.

Afatinib is a tyrosine kinase receptor inhibitor that binds to the kinase domains of EGFR and HER2 with IC50 of 0.5 nM and 14 nM, respectively [28]. With the working concentration we used for Afatinib (1 nM), we would expect it to work solely as an inhibitor of EGFR tyrosine phosphorylation. We did not include the “CPV+Afatinib” group in the study design as is it difficult to interpret the results from combined treatment of CPV and Afatinib. This might be a resultant from inconsistent effects of CAP on different EGFR phosphorylation sites. Specifically, adding CPV would decrease EGFR (Y1068) and increase EGFR (Y1086) phosphorylation, adding Afatinib decreased both EGFR (Y1086) and EGFR (Y1068) phosphorylation; thus, exposing cells to both CPV and Afatinib would down-regulate EGFR (Y1068) phosphorylation for sure but increase/decrease EGFR (Y1086) phosphorylation depending on which effect (CPV or Afatinib) dominated. Thus, the outcomes from the “CPV+Afatinib” group could resemble any of the other three groups, i.e. control, “CPV,” and “Afatinib,” and vary in every experiment that contributes nothing but complications. In addition, we did not examine cells’ response to the modulation of EGFR expression given the involvement of a reversed phosphorylation pattern between EGFR (Y1086) and EGFR (Y1068) in triggering cell cycle arrest.

The cytoplasmic domain of EGFR has five functional EGFR phosphorylation sites (i.e. Y1068, Y1086, Y1148, Y992, and Y1173) that are important for downstream signaling and are required for mitogenic responses to EGFR activation [29]. EGFR phosphorylation sites Y992 and Y1173 play critical roles in activating the MAPK cascade following EGF stimulation [30]. Given reduced cell viability (Figure 1(b)) on CPV infection and the prominent role of MAPK signaling in regulating cell proliferation, we did not focus on these two sites but rather focused on EGFR Y1068, Y1086, Y1148 sites, and found that phosphorylation of the EGFR Y1086 site mediated the G1/S cell cycle arrest. We could not exclude the possibility that other Tyrosine phosphorylation sites, such as Y845 and Y1101 that function in response to EGFR activation, are also involved in this process. Also, EGFR phosphorylation is not limited to Tyrosine, other amino acids such as serine and threonine residues in EGFR might also be phosphorylated, which include a PKC site (T654), 4 CAMKII sites (S1046, S1047, S1057, S1142) and two constitutively phosphorylated sites (S967, S1002). Whether and how do these sites contribute to CPV-induced G1/S cell cycle arrest remain to be elucidated. In addition, we demonstrated in this study, the involvement of differential phosphorylation status of different EGFR phosphorylation sites in G1/S arrest on CPV infection but did not mutate these specific phosphorylation sites to directly prove their roles in halting cell cycle. These together constitute our future studies.

Excessive intracellular ROS in response to viral infection could regulate many cellular signalings including cell cycle through modulating the phosphorylation status of growth factor receptors such as EGFR [31]. Compared with EGF that stimulates phosphorylation of EGFR at both S/T and Y sites, hydrogen peroxide (a key component of ROS) preferentially triggers EGFR Y phosphorylation [32]. Therefore, we would presume that EGFR could be activated by elevated intracellular ROS in response to viral infection, and establishing pseudoviruses (i.e. inactivated viruses) targeting malignant cells may arrest cancer cells at certain cell cycle stages through the same mechanism for the sake of therapeutics.

Though not many, several studies have reported parvovirus-induced cell cycle arrest that was considered to favor viral replication. These include minute virus of mice (MVM) elicited G1/S arrest via activated ATM (ataxia telangiectasia mutated protein)-mediated DNA damage repair signaling [10–12,14], and parvovirus H-1 (H-1PV) triggered G2/M arrest that is less well characterized but considered associated with the accumulation of reactive oxygen species [13,14]. This study, for the first time, elucidated the role of EGFR and its phosphorylation site Tyr1086 in triggering cell cycle arrest at the G1/S stage in CPV. Our results avail in the development of virotherapies utilizing CPV against cancers given the functionalities of EGFR in regulating TfR that plays dual roles in CPV entry and Fenton effect as well as in triggering host cell cycle arrest as unveiled here.

## Conclusion

We report in this study that CPV could arrest host cells at the G1/S stage to favor its multiplication, and this process occurs in response to EGFR (Y1086) phosphorylation and EGFR (Y1068) de-phosphorylation through the EGFR (Y1086)/p27 and STAT3 (Y705)/cyclin D1 axes. Our results not only contribute to the understanding of CPV-triggered cell cycle arrest but also suggest one mechanism that can be used to prevent cell cycle arrest to add in the appropriate design of parvovirus-mediated virotherapies against cancers.

## Supplementary Material

Supplemental Material
