# Supplementary material for: Canine parvovirus induces G1/S cell cycle arrest that involves EGFR Tyr1086 phosphorylation
Source: Virulence. 2020 Sep 2;11(1):1203–14. doi: 10.1080/21505594.2020.1814091 (PMC7549965; doi:10.1080/21505594.2020.1814091)
Supplement: Supplemental Material [file KVIR_A_1814091_SM9217.pptx]

## Slide 1
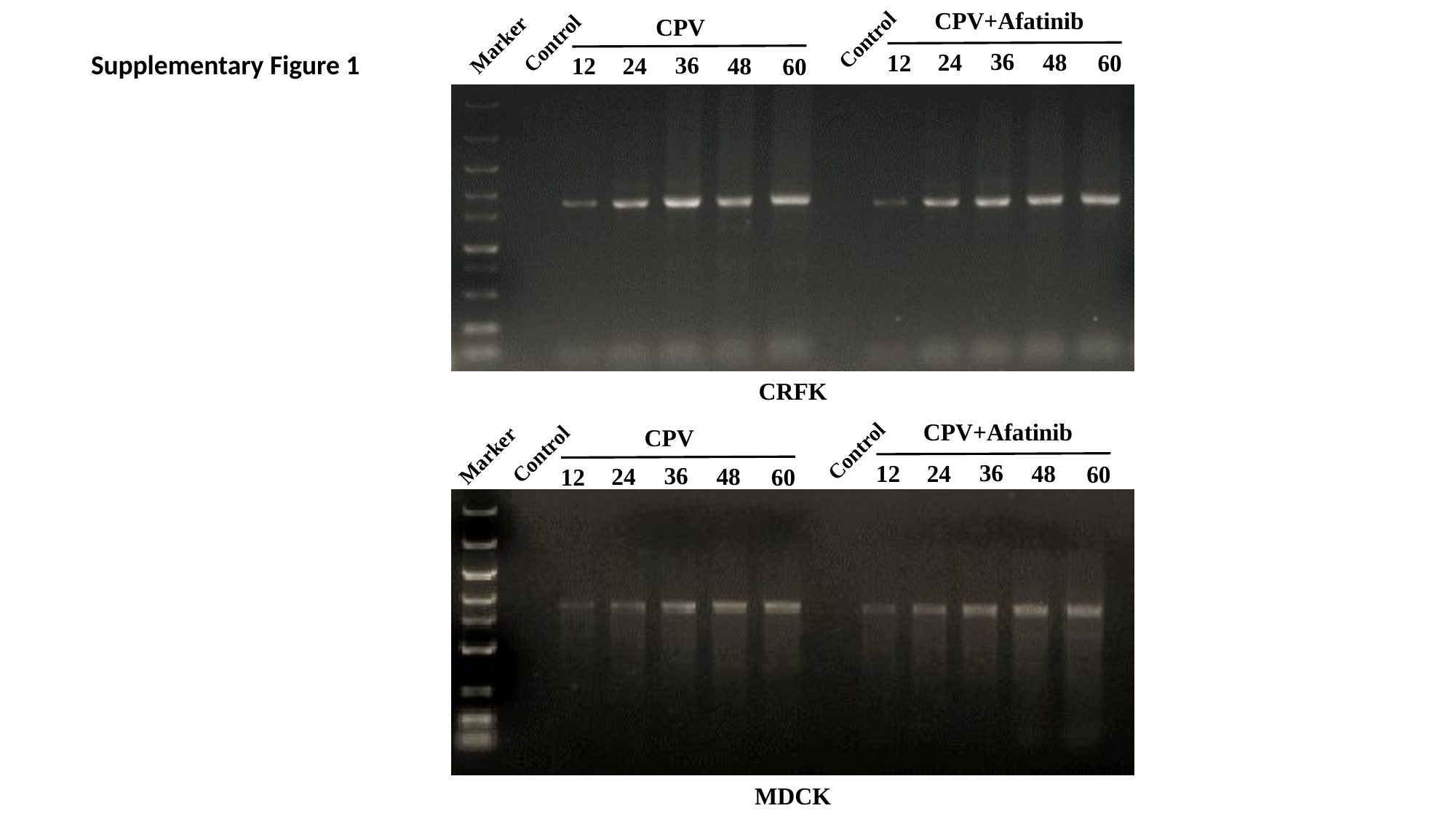

CPV+Afatinib
Control
36
24
48
12
60
CPV
Control
Marker
36
24
48
12
60
CRFK
CPV+Afatinib
Control
36
24
48
12
60
CPV
Control
Marker
36
24
48
12
60
MDCK
Supplementary Figure 1

## Slide 2
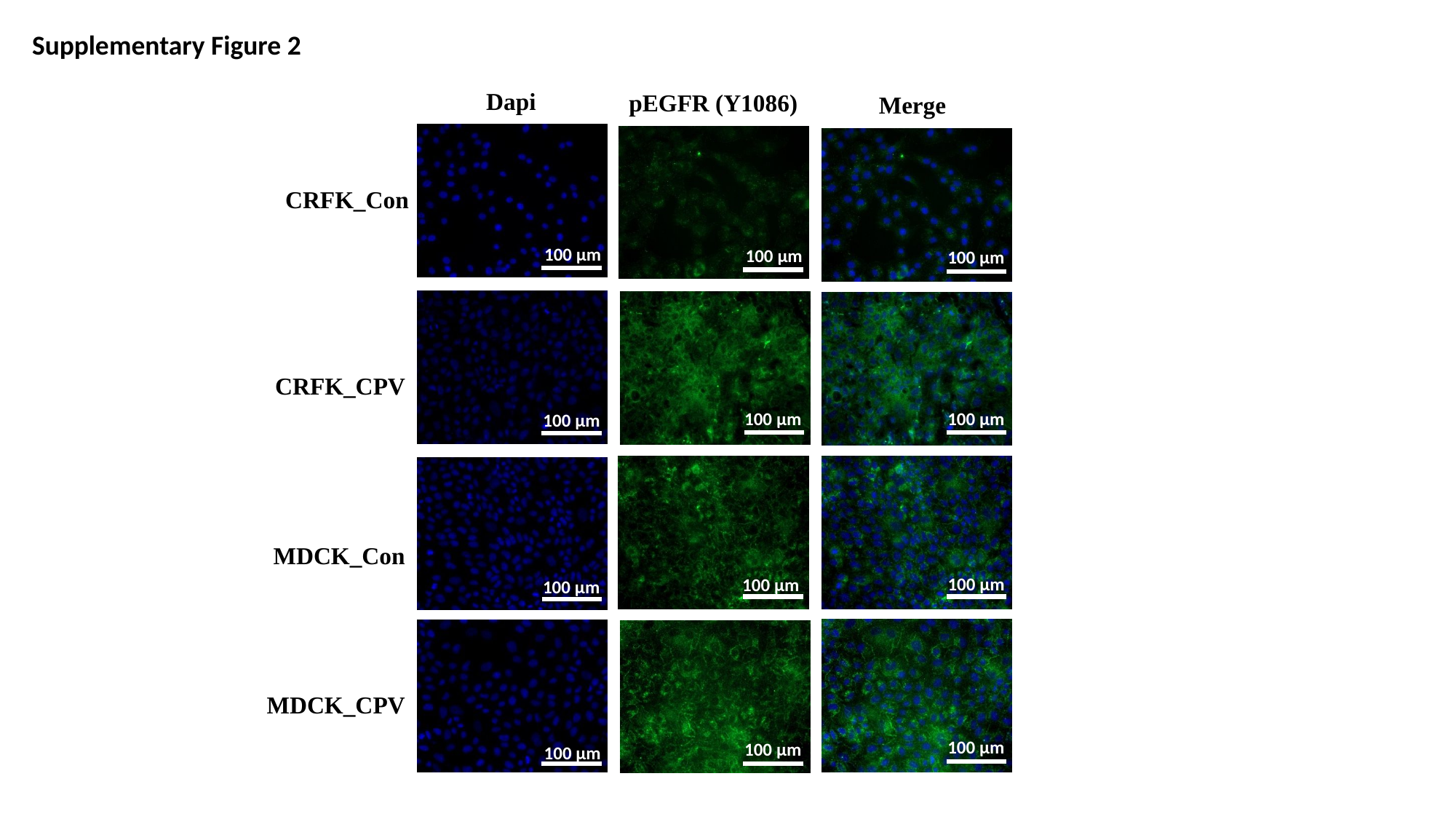

Supplementary Figure 2
Dapi
pEGFR (Y1086)
Merge
CRFK_Con
100 μm
100 μm
100 μm
100 μm
100 μm
100 μm
100 μm
100 μm
100 μm
100 μm
100 μm
100 μm
CRFK_CPV
MDCK_Con
MDCK_CPV

## Slide 3
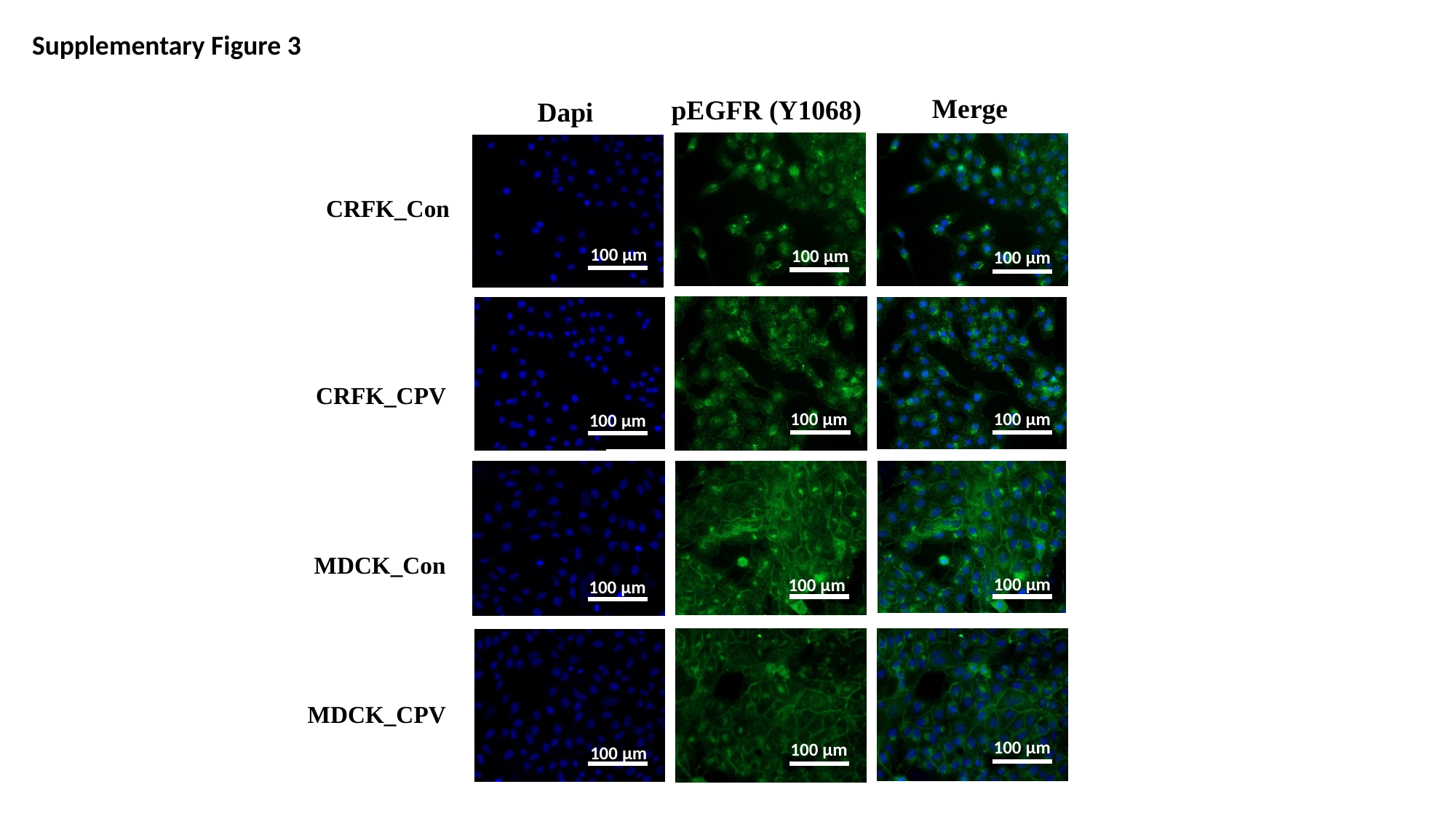

Supplementary Figure 3
Merge
pEGFR (Y1068)
Dapi
CRFK_Con
100 μm
100 μm
100 μm
100 μm
100 μm
100 μm
100 μm
100 μm
100 μm
100 μm
100 μm
100 μm
CRFK_CPV
MDCK_Con
MDCK_CPV

## Slide 4
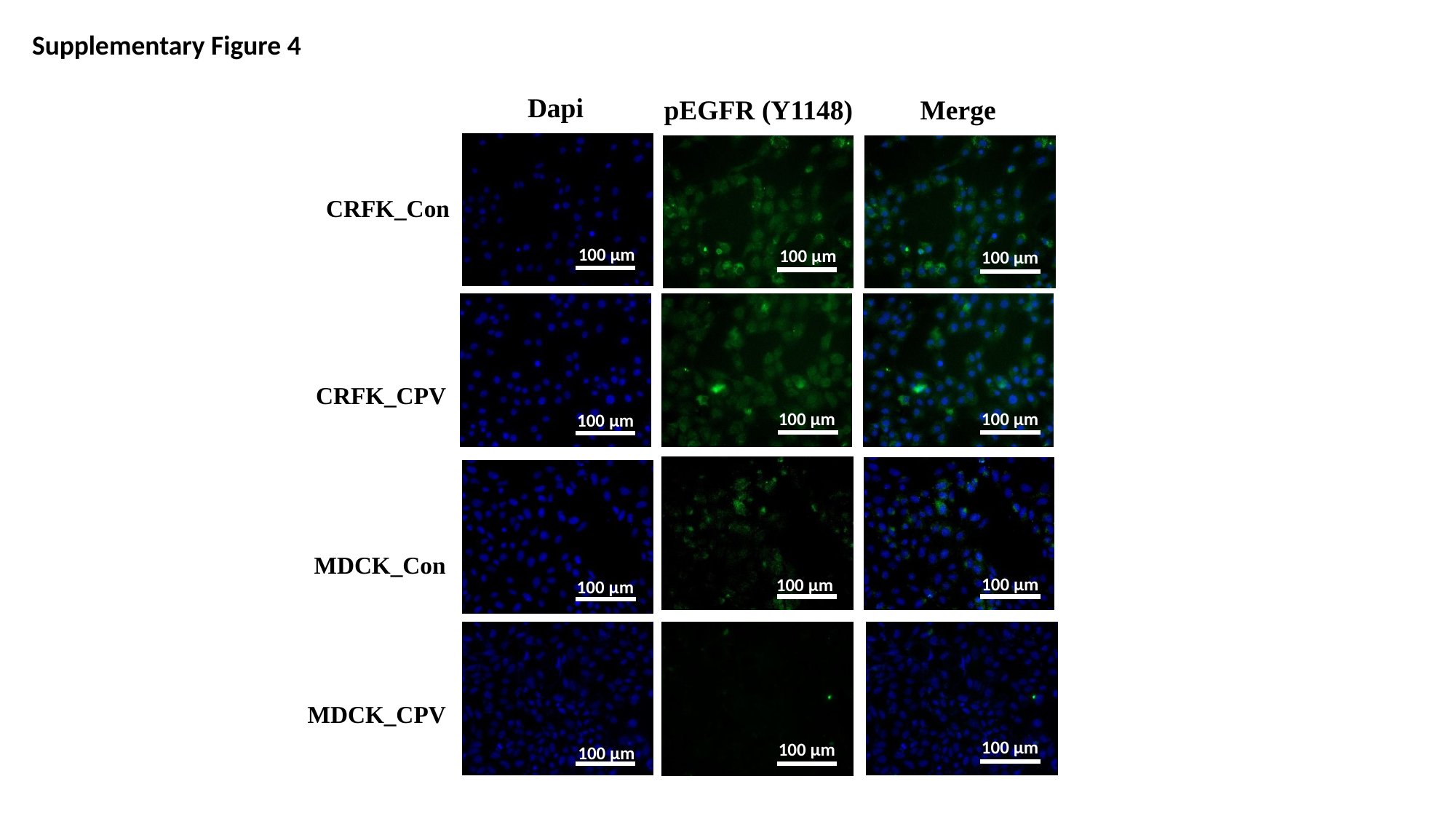

Supplementary Figure 4
Dapi
pEGFR (Y1148)
Merge
CRFK_Con
100 μm
100 μm
100 μm
100 μm
100 μm
100 μm
100 μm
100 μm
100 μm
100 μm
100 μm
100 μm
CRFK_CPV
MDCK_Con
MDCK_CPV
